# Supplementary material for: Honeybee Pollen Extracts Reduce Oxidative Stress and Steatosis in Hepatic Cells
Source: Molecules. 2020 Dec 22;26(1):6. doi: 10.3390/molecules26010006 (PMC7792600; doi:10.3390/molecules26010006)
Supplement: Supplementary file 1 [file molecules-26-00006-s001.pdf]

Supplementary Materials

Honeybee Pollen Extracts Reduce Oxidative Stress and Steatosis in Hepatic Cells

Juan Esteban Oyarzún [1], Marcelo E. Andia [1], Sergio Uribe [1], Paula Núñez Pizarro [2], Gabriel Núñez [3], Gloria Montenegro [3] and Raquel Bridi [2,*]

[1] Biomedical Imaging Center, School of Medicine, Pontificia Universidad Católica de Chile, Chile, and ANID-Millennium Science Initiative Program-Millennium Nucleus for Cardiovascular Magnetic Resonance, Santiago, Chile; jeoyarzu@uc.cl (J.E.O.); meandia@uc.cl. (M.E.A.); suribe@uc.cl (S.U.)
[2] Departamento de Farmacia, Facultad de Química, Pontificia Universidad Católica de Chile, Avda Vicuña Mackenna 4860, Macul, Santiago, Chile; pjnunez@uc.cl
[3] Departamento de Ciencias Vegetales, Facultad de Agronomía e Ingeniería Forestal, Pontificia Universidad Católica de Chile, Avda Vicuña Mackenna 4860, Santiago, Chile; ginunez@uc.cl (G.N.); gmonten@uc.cl (G.M.)
* Correspondence: rbridi@uc.cl

Table S1. Botanical origin and classification of bee pollen samples. Data is represented as the percentage of participation of each species in the total sample, according to weight of grams of pollen loads.

2018									
**Sample**	**Clasification**	**Predominant Species (≥45%)**		**Secondary Species (16-45%)**		**Important Minor Species (3-15%)**		**Minor Species (≤3%)**	
**1 (65)**	Multifloral Non-Native					*Eschscholzia californica*<br>*Adesmia arbórea*<br>*Taraxacum officinale*<br>*Cryptocarya alba*<br>*Peumus boldus*<br>*Schinus molle*<br>*Sophora macrocarpa*<br>*Brassica rapa*<br>*Raphanus sativus*	15.41<br>15.13<br>12.89<br>10.92<br>10.92<br>9.81<br>8.96<br>8.96<br>3.92	*Cuscuta chilensis*<br>*Fungus Spores*	2.24<br>0.84
**2 (37)**	Multifloral Non-Native			*Eschscholzia californica*<br>*Brassica rapa*<br>*Eucalyptus sp*	37.42<br>22.7<br>16.36	*Anthemis cotula*<br>*Carduus sp.*<br>*Schinus latifolius*	9.61<br>6.54<br>5.52	*Cissus striata*	1.84
**3 (67)**	Multifloral Non-Native			*Brassica rapa*	37	*Cryptocarya alba*<br>*Sophora macrocarpa*<br>*Cactaceae*<br>*Adesmia arbórea*<br>*Peumus boldus*<br>*Eschscholzia californica*<br>*Taraxacum officinale*	14.2<br>11.2<br>11<br>9.4<br>7.4<br>5.8<br>4		
**4 (53)**	Monofloral Non-Native	*Brassica rapa*	51.12	*Cryptocarya alba*	17.94	*Anthemis cotula*<br>*Peumus boldus*<br>*Eschscholzia californica*<br>*Raphanus sativus*	11.66<br>7.85<br>6.05<br>5.38		
**5 (38)**	Monofloral Non-Native	*Brassica rapa*	46.09	*Adesmia arbórea*	21.14	*Colliguaja odorifera*<br>*Eschscholzia californica*<br>*Taraxacum officinale*<br>*Schinus latifolius*<br>*Azara celastrina*	8.67<br>7.82<br>7.61<br>5.49<br>3.17		
**6 (32)**	Multifloral Native			*Acacia caven*<br>*Eschscholzia californica*	41,64<br>18.22	*Brassica rapa*<br>*Hypochaeris radicata*<br>*Baccharis linearis*<br>*Aextoxicon punctatum*	15.43<br>9.29<br>6.13<br>6.13	*Schinus latifolius*<br>*Colliguaja odorifera*	2.97<br>0.19
**7 (56)**	Monofloral Non-Native	*Brassica rapa*	47.11	*Cryptocarya alba*	24.89	*Azara petiolaris*<br>*Eschscholzia californica*<br>*Hypochaeris radicata*	14.22<br>7.78<br>6		
**8 (55)**	Multifloral Non-Native			*Brassica rapa*	42.66	*Azara petiolaris*	14.45		

				Cryptocarya alba	18.28	*Eschscholzia californica*<br>*Hypochaeris radicata*	14.45<br>10.16		
**9 (35)**	Monofloral<br>Non-Native	*Brassica rapa*	65.39	*Colliguaja odorifera*<br>*Amomyrtus luma*	17.3<br>16.10			*Baccharis linearis*	1.21
**10 (51)**	Monofloral<br>Non-Native	*Brassica rapa*	53.73	*Eschscholzia californica*	21.93	*Cryptocarya alba*<br>*Trichocereus chilensis*<br>*Maytenus boaria*	7.9<br>5.7<br>5.48	*Acacia caven*<br>*Anthemis cotula*<br>*Foeniculum vulgarus*	2.85<br>1.97<br>0.44
**11 (39)**	Multifloral Non-Native			*Azara celastrina*<br>*Non identified*	23.83<br>19.34	*Cryptocarya alba*<br>*Eschscholzia californica*<br>*Brassica rapa*<br>*Kageneckia oblonga*<br>*Cactaceae*	15.43<br>14.45<br>10.94<br>8.01<br>6.25	*Convolvulus arvensis*	1.76
**12 (42)**	Multifloral<br>Non-Native			*Cryptocarya alba*<br>*Brassica rapa*	36.15<br>25.97	*Eschscholzia californica*<br>*Adesmia arbórea*<br>*Eucalyptus sp.*<br>*Sophora macrocarpa*<br>*Lomatia hirsuta*	11.26<br>8.66<br>8.23<br>5.2<br>4.11	*Carduus pycnocephalus*	0.22
**13 (33)**	Monofloral<br>Non-Native	*Brassica sp.*	89.29			*Colliguaja odorifera*<br>*Eschscholzia californica*	4.37<br>3.57	*Raphanus sativus*<br>*Hypochaeris radicata*	1.57<br>1.19
**14 (40)**	Multifloral<br>Non-Native			*Brassica rapa*<br>*Adesmia arbórea*	30.03<br>16.71	*Cryptocarya alba*<br>*Schinus latifolius*<br>*Convolvulus arvensis*<br>*Azara celastrina*	15.41<br>13.06<br>10.97<br>10.18	*Anthemis cotula*	3.66
**15 (63)**	Multifloral<br>Non-Native					*Brassica rapa*<br>*Eucalyptus sp.*<br>*Raphanus sativus*<br>*Schinus latifolius*<br>*Scolymus sp.*<br>*Adesmia arborea*<br>*Rosaceae*<br>*Cactaceae*<br>*Maytenus boaria*<br>*Epilobium sp.*<br>*Acacia melanoxylon*<br>*Fungus spores*<br>*Fern spores*<br>*Azara petiolaris*	14.64<br>10.63<br>8.42<br>7.49<br>7.15<br>6.68<br>6.05<br>5.42<br>5.2<br>4.99<br>4.91<br>4.87<br>4.82<br>4.69<br>4.65		
**16 (62)**	Multifloral<br>Non-Native			*Brassica rapa*<br>*Cryptocarya alba*	33.33<br>18.12	*Hypochaeris radicata*<br>*Raphanus sativus*<br>*Schinus latifolius*<br>*Adesmia arbórea*<br>*Carduus sp.*	13.53<br>9.18<br>7.49<br>7.25<br>6.28		

						Anthemis cotula	4.83		
**17 (61)**	Monofloral<br>Non-Native	*Brassica rapa*	59.45			*Cryptocarya alba*<br>*Schinus latifolius*<br>*Hypochaeris radicata*<br>*Adesmia arbórea*<br>*Eschscholzia californica*	14.75<br>9.22<br>5.76<br>4.84<br>3.92	*Carduus pycnocephalus*	2.07
**18 (31)**	Multifloral<br>Non-Native			*Eschscholzia californica*<br>*Brassica rapa*	41.45<br>34.21	*Schinus latifolius*<br>*Medicago sativa*	11.27<br>7.24	*Trichocereus chilensis*<br>*Galega oficinalis*	2.62<br>1.21
**19 (36)**	Multifloral Non-Native			*Cryptocarya alba*<br>*Brassica rapa*	36.61<br>35.7	*Eschscholzia californica*<br>*Raphanus sativus*<br>*Taraxacum officinale*	12.2<br>8.19<br>7.1	*Eucalyptus globulus*	0.18
**20 (57)**	Multifloral Non-Native			*Eschscholzia californica*<br>*Cryptocarya alba*<br>*Anthemis cotula*	39.61<br>24.03<br>17.32	*Fumaria officinalis*<br>*Unidentifies pollen*	10.17<br>6.93	*Acacia caven*	1.95
**21 (52)**	Multifloral<br>Non-Native			*Brassica rapa*<br>*Lythrum hyssopifolia*<br>*Robinia pseudoacacia*<br>*Eschscholzia californica*	27.14<br>20.88<br>16.49<br>15.87	*Cryptocarya alba*<br>*Schinus latifolius*<br>*Anemone nemorosa*<br>*Maytenus boaria*	6.89<br>3.97<br>3.76<br>3.76	*Azara celastrina*	1.25
**22 (43)**	Multifloral<br>Non-Native			*Brassica rapa*<br>*Eschscholzia californica*	38.29<br>23.42	*Cactaceae*<br>*Adesmia arbórea*<br>*Schinus latifolius*<br>*Trichocereus chiloensis*	12.39<br>11.29<br>8.26<br>3.31	*Anthemis cotula*<br>*Carduus pycnocephalus*	2.2<br>0.83
**23 (54)**	Monofloral<br>Native	*Cryptocarya alba*	45.04	*Eschscholzia californica*	20.66	*Taraxacum officinale*<br>*Foeniculum vulgare*<br>*Schinus latifolius*<br>*Acacia caven*<br>*Anthemis cotula*	8.47<br>7.65<br>7.44<br>5.37<br>3.1	*Brassica rapa*<br>*Luma apiculata*	1.45<br>0.83
**24 (41)**	Monofloral<br>Non-Native	*Brassica rapa*	45.23			*Schinus latifolius*<br>*Azara celastrina*<br>*Taraxacum officinale*<br>*Raphanus sativus*<br>*Oenothera sp.*<br>*Cuscura chilensis*	13.69<br>11.74<br>9.05<br>7.58<br>4.89<br>3.18	*Anthemis cotula*<br>*Carduus sp.*	2.45<br>2.2
**25 (60)**	Multifloral<br>Non-Native			*Brassica rapa*<br>*Cryptocarya alba*<br>*Azara petiolaris*	29.07<br>25.35<br>20.47	*Hypochaeris radicata*<br>*Eschscholzia californica*<br>*Carduus sp.*	10.47<br>8.84<br>3.49	*Cuscuta chilensus*	2.33
**26 (45)**	Monofloral<br>Native	*Cryptocarya alba*	56.58			*Eschscholzia californica*<br>*Malva sp.*<br>*Cactaceae*<br>*Anthemis cotula*	11.4<br>10.31<br>7.24<br>6.36	*Acacia caven*<br>*Crataegus monogyna*<br>*Maytenus boaria*	2.41<br>1.75<br>0.22

						Dicliptera sp.	3.73		
**27 (44)**	Monofloral Native	*Cryptocarya alba*	45.67			*Eschscholzia californica*	13.88	*Brassica rapa*	2.62
						Anthemis cotula	11.07		
						Schinus polygamus	8.25		
						Carduus sp.	6.24		
						Schinus latifolius	4.63		
						Acacia caven	4.43		
						Fabaceae nn	3.22		
**28 (58)**	Multifloral Non-Native			*Cryptocarya alba*	29.47	*Baccharis linearis*	11.83		
				Anthemis cotula	18.34	*Cactaceae nn*	10.58		
						Colliguaja odorifera	10.42		
						Maytenus boaria	9.95		
						Schinus latifolius	9.4		
